# A Case of Neuroleptic Malignant Syndrome in a Profoundly Intellectually Disabled Patient with Successful Reintroduction of Antipsychotic Therapy with Quetiapine

**DOI:** 10.1155/2018/7045106

**Published:** 2018-07-15

**Authors:** Kamal Patel, Brandon Lilly, Oluwadamilare Ajayi, Kelly Melvin

**Affiliations:** Department of Psychiatry and Behavioral Medicine, Marshall University Joan C. Edwards School of Medicine, Huntington, WV 25701, USA

## Abstract

Neuroleptic Malignant Syndrome (NMS) is a rare condition clinically characterized by muscle rigidity, hyperthermia, autonomic instability, and acute mental status change. NMS is most often associated with use of high-potency first-generation antipsychotic medications; though, other neuroleptics have been implicated as well. NMS can be fatal with estimated mortality rates as high as 20%. Patients experiencing certain severe complications, including renal failure, have been associated with mortality as high as 50%, stressing the need for early recognition and treatment. Here we present the case of a 54-year-old male that initially presented with symptoms suspicious for sepsis, but who eventually developed a clinical picture consistent with NMS. We describe the diagnostic and treatment process leading to symptom remission. We then discuss our decision to reintroduce an atypical antipsychotic agent, quetiapine. This case illustrates the importance of early recognition of the signs and symptoms of NMS and the need to initiate treatment promptly in order to prevent complications, including death. This case also highlights the decision to resume antipsychotic pharmacotherapy after adequate resolution of NMS, demonstrating that it can be done so safely if started at low doses coupled with intensive monitoring of the patient.

## 1. Introduction

NMS is a rare condition clinically characterized by muscle rigidity, hyperthermia, autonomic instability, and acute mental status change with a current incidence rate ranging from 0.01 to 0.02% [1]. NMS is most often associated with use of neuroleptic medications such as antipsychotics. Typically, high-potency first-generation antipsychotics such as trifluoperazine and haloperidol are frequently associated with NMS as compared to the second-generation antipsychotics [1, 2]. Mortality rates secondary to NMS varied, but recent estimates place them at 10% [1]. Illness severity and complications such as renal failure predict higher mortality. Rechallenge with atypical agents has been done in the past [3]; however, there are scarce records of rechallenge with quetiapine. Of the few cases with quetiapine rechallenge after NMS, results are conflicting [4, 5]. Here we discuss a case of NMS, its treatment, and subsequent successful rechallenge with the atypical agent quetiapine.

## 2. Case Presentation

A 54-year-old Caucasian male with past medical history of profound intellectual disability, schizophrenia, posttraumatic stress disorder, Parkinson's disease, gastroesophageal reflux disease, and seizure disorder initially presented to the emergency department (ED) from an assisted living facility with fever, tachycardia, nausea, and vomiting of three days duration. The patient was diagnosed with sepsis of unknown origin and admitted to the hospital for further workup and treatment. On admission, vitals showed the following: temperature of 102.5F, pulse 123 beats per minute, respiratory rate 40 breaths per minute, O2 saturation of 96% on room air, and blood pressure 154/84 mm/Hg. On imaging, chest X-ray showed no evidence of acute cardiopulmonary disease. Electrocardiogram showed sinus tachycardia with possible right atrium enlargement and ventricle hypertrophy. Laboratory studies showed the patient had no leukocytosis; however, segmented neutrophil was 92.8% and absolute neutrophil count was 9.19 k/cmm. Hemoglobin and hematocrit were decreased at 12.8 and 38.0%, respectively. Atrial blood gas was unremarkable. Lactic acid sepsis was initially 1.96 mmol/L and later increased to 2.63 mmol/L. Procalcitonin was 0.32 ng/ml. Metabolic panel showed sodium was 149, chloride 114, BUN 37 mg/dL, AST 136, ALT 43. Urinalysis showed trace blood likely secondary to catheterization and urine ketones of 40 mg/dL. Urine culture was negative after 48 hours. Blood cultures from admission were negative after five days. Influenza screen was negative. Viral PCR was negative. Physical examination at admission showed the patient was in moderate distress, diaphoretic, agitated, tachycardic, and tachypneic. The patient was started on antibiotic therapy with vancomycin, levofloxacin, and piperacillin/tazobactam for sepsis of unknown origin and given fluid resuscitation. Home medications included the following: paliperidone 3mg daily, haloperidol 2.5mg twice daily as needed, citalopram 40mg daily, carbidopa/levodopa 25-100mg three times daily, and carbamazepine ER 200mg twice daily.

On hospital day 2, the patient developed increased muscle rigidity, tremors, hypoxemia with O2 saturation in the 70s on room air, increasing temperature (102.9F), tachypnea (max respiratory rate 45), and worsening tachycardia (max heart rate 130). Repeat labs revealed leukocytosis with a white cell count of 11.9 k/cmm and neutrophil count of 89.6 k/cmm. Patient also developed acute renal insufficiency with BUN and creatinine of 52 mg/dL and 1.53 mg/dL, respectively. Lactic acid further increased to 4.3 mmol/L. Total creatine kinase was 2249 u/L.

The patient was transferred to intensive care unit (ICU), intubated, and ventilated for airway protection. Close review of medical records from his assisted living facility indicated that the patient had received intramuscular injections of haloperidol 2.5mg seven times over the previous three weeks to help with behavioral agitation. The last dose was administered on the day of admission. Over the same period of time, it also appeared that the patient had been refusing many of his scheduled medications to include carbidopa/levodopa. The patient's history, examination, and laboratory findings strongly suggested a diagnosis of NMS and treatment was initiated accordingly. A one-time dose of dantrolene 125mg IV was given. Additionally, the patient received supportive care measures including application of a cooling blanket and infusion of cooled normal saline provided for hyperpyrexia. Intravenous vecuronium was started for severe muscle rigidity. All psychotropic agents were held.

Neurology recommended video electroencephalography (VEEG) due to the patient's history of seizures. VEEG showed no epileptiform activity and mild nonspecific encephalopathy. Neurology recommended the resumption of carbidopa/levodopa 37.5-150mg three times daily and carbamazepine 200mg twice daily with titration to a final dose of 500mg twice daily with a target carbamazepine level of 10-12. Throughout the ICU stay, the patient's total CK level trended downward, decreasing from 2249 u/L to 535 u/L. BUN and creatinine trended down to 23 mg/dL and 0.85 mg/dL.

As the patient's condition improved, he was extubated and transferred to the general medicine unit. However, with resumption of carbidopa/levodopa, the patient's untreated psychotic symptoms exacerbated and included audio-visual hallucinations and delusional paranoia. He was also intermittently agitated and not sleeping well. Psychiatry was consulted for recommendations regarding treatment of psychosis in the setting of resolving NMS. Carbamazepine and lorazepam were recommended for management of agitation, and trazodone 100mg at bedtime was started for sleep. Psychotic symptoms and agitation persisted despite being on lorazepam 2mg three times daily as well as being on a therapeutic dosage of carbamazepine. The decision was made to start low dose quetiapine at 12.5mg at bedtime and titrated up to 12.5mg twice daily after four days, at which point the patient's psychosis diminished. The patient was monitored closely throughout for recurrence of NMS symptoms. Quetiapine was well-tolerated and the patient demonstrated improvement in psychosis, agitation, sleep, and appetite. After X days, the patient returned to his baseline mentation and was successfully discharged back to his assisted living facility.

## 3. Discussion

This case illustrates many of the complexities involved in diagnosis and management of NMS. Diagnostically, NMS can be challenging to recognize without a high index of clinical suspicion. The illness is often mistaken for an infectious condition such as sepsis or for similar disorders of autonomic regulation including serotonin syndrome or malignant catatonia. There are no universally agreed upon criteria for diagnosing NMS due to variable clinical presentations [11]. However, it is thought that the tetrad of altered mental status, hyperthermia, muscle rigidity, and autonomic instability comprise the illness' core symptomatology (Table 1) [6–10]. These symptoms, along with known exposure to a neuroleptic agent, should raise suspicion for treating clinicians.

Our case further illustrates the importance of early recognition of NMS and the need to promptly initiate therapy to avoid severe complication such as multistate organ failure, which may subsequently result in death [12].

In our case, the patient experienced all four cardinal symptoms. He had positive exposure in the form of scheduled paliperidone, a second-generation antipsychotic, as well as haloperidol, a high-potency first-generation antipsychotic. Further, our patient's refusal of his medications during the weeks leading up to admission led to abrupt withdrawal of his carbidopa/levodopa, a dopamine agonist. Abrupt reduction or cessation of dopaminergic agent is at the core of the development of NMS [13, 14]. There have been several reports in which withdrawal of carbidopa/levodopa has precipitated NMS or an NMS-like condition [13, 15]. In the case of our patient, both factors likely played a role in the development of his symptoms.

Finally, the decision of when to, or whether to, resume antipsychotic treatment for NMS patients can be challenging. The estimated risk of recurrence is approximately 30% [9, 16]. Given the risk of recurrence and the associated NMS mortality rate, the clinician and patient must carefully weigh the potential risks of treatment against those of poorly controlled psychiatric illness. Nevertheless, antipsychotic agents can be reinitiated safely after adequate resolution of NMS symptoms. There is some evidence to suggest that second-generation antipsychotics may present a lower risk, especially if started at low doses, gradually titrated, and coupled with intensive patient monitoring [16–18]. They are not, however, completely protective as there are cases of NMS in the literature secondary to therapy with quetiapine and other second-generation antipsychotics [3, 17].

As stated previously, the literature on quetiapine rechallenge after NMS is conflicting [4, 5]. Hatch et al. in their case unsuccessfully rechallenged a patient who had developed NMS symptoms secondary to typical antipsychotic therapy (haloperidol and chlorpromazine). Following resolution of their patient's NMS symptoms, a dose of quetiapine 25mg twice daily was introduced, which resulted in a resurfacing of NMS [5]. Conversely, Tripathi et al. in their case had a patient with diagnosis of NMS while on olanzapine 20mg daily. Olanzapine was discontinued and started on quetiapine which was titrated up to 500mg daily over 3 weeks following complete NMS resolution without symptom relapse [19].

Our case illustrates successful reintroduction of much needed antipsychotic therapy following remission of NMS symptoms. Quetiapine was introduced cautiously at a low dose (12.5mg) and slowly titrated to a favorable symptom response. The patient was evaluated daily during hospitalization and examined for any recurrence of NMS symptoms. Regarding risk evaluation, the severity of untreated symptoms was causing agitated aggression and interfering with his medical care. After discussion of potential risks and benefits with his medical decision maker, we were able to initiate treatment that fortunately resulted in a positive outcome.

## 4. Conclusion

This case highlights the importance of early recognition and treatment for NMS. Additionally, our case contributes to the literature supporting successful reintroduction of antipsychotic therapy. Specifically, our patient successfully tolerated quetiapine at low dose following resolution of NMS under careful monitoring.

## Figures and Tables

**Table 1:** Tetrad or cardinal features of NMS [6–10].

**Typical Symptoms**	**Prevalence in NMS**
Acute Mental Status Changes	82%
Severe Muscle Rigidity	45-92%
Hyperthermia	87% (38C), 40% (40C)
Autonomic Instability	88% (tachycardia), 61-77% (labile or high BP), 73% (Tachypnea)
